# Identification of novel alternative splicing associated with mastitis disease in Holstein dairy cows using large gap read mapping

**DOI:** 10.1186/s12864-022-08430-x

**Published:** 2022-03-19

**Authors:** V. Asselstine, J. F. Medrano, A. Cánovas

**Affiliations:** 1 Centre for Genetic Improvement of Livestock, Department of Animal Biosciences, University of Guelph, 50 Stone Road East, Building #70, Guelph, ON N1G 2W1 Canada; 2 Department of Animal Science, University of California – Davis, Davis, CA 95616 USA

**Keywords:** Dairy cattle, Large gap read mapping, Mastitis, Milk somatic cell, RNA - Sequencing, Alternative splicing, QTL

## Abstract

**Background:**

Mastitis is a very common disease in the dairy industry that producers encounter daily. Transcriptomics, using RNA-Sequencing (RNA - Seq) technology, can be used to study the functional aspect of mastitis resistance to identify animals that have a better immune response to mastitis. When the cow has mastitis, not only genes but also specific mRNA isoforms generated via alternative splicing (AS) could be differentially expressed (DE), leading to the phenotypic variation observed. Therefore, the objective of this study was to use large gap read mapping to identify mRNA isoforms DE between healthy and mastitic milk somatic cell samples (*N* = 12). These mRNA isoforms were then categorized based on being 1) annotated mRNA isoforms for gene name and length, 2) annotated mRNA isoforms with different transcript length and 3) novel mRNA isoforms of non - annotated genes.

**Results:**

Analysis identified 333 DE transcripts (with at least 2 mRNA isoforms annotated, with at least one being DE) between healthy and mastitic samples corresponding to 303 unique genes. Of these 333 DE transcripts between healthy and mastitic samples, 68 mRNA isoforms are annotated in the bovine genome reference (ARS.UCD.1.2), 249 mRNA isoforms had novel transcript lengths of known genes and 16 were novel transcript lengths of non - annotated genes in the bovine genome reference (ARS.UCD.1.2). Functional analysis including gene ontology, gene network and metabolic pathway analysis was performed on the list of 288 annotated and unique DE mRNA isoforms. In total, 67 significant metabolic pathways were identified including positive regulation of cytokine secretion and immune response. Additionally, numerous DE novel mRNA isoforms showed potential involvement with the immune system or mastitis. Lastly, QTL annotation analysis was performed on coding regions of the DE mRNA isoforms, identifying overlapping QTLs associated with clinical mastitis and somatic cell score.

**Conclusion:**

This study identified novel mRNA isoforms generated via AS that could lead to differences in the immune response of Holstein dairy cows and be potentially implemented in future breeding programs.

**Supplementary Information:**

The online version contains supplementary material available at 10.1186/s12864-022-08430-x.

## Background

Mastitis is a very common inflammatory disease that dairy producers face in their herds. Although mastitis has been studied in terms of its impact on milk yield, composition, health and welfare of the animal, studying the genetic mechanisms that make some cows better able to adapt to mastitis has not been studied thoroughly [1]. Studying the bovine transcriptome provides important information at the host level, however, using a cost effective and minimally invasive technique to obtain this information is critical. Next generation sequencing technologies have advanced this area for high - throughput functional genomics [2, 3].

One high - throughput technology, RNA - Sequencing (RNA - Seq), characterizes the transcriptome of the host and has been developed to identify and quantify tissue - specific genes and transcripts that are differentially expressed (DE [4]). RNA - Sequencing can also be used to identify DE alternative splicing (AS) variants and structural variation in the coding region such as SNPs and INDELs [5–8]. The phenomenon of AS plays an important role in regulating the mammalian proteome and disease processes [9], as AS generates different mature transcripts from the same primary RNA sequence [10, 11] from a specific gene [12]. These mRNA isoforms can then be translated into functionally similar proteins that have similar but not identical amino acid sequences that contribute to observed phenotypic variation [12].

Previous research has investigated novel isoforms derived by AS and found that the isoform levels were DE in healthy and infected mammary tissues [11]. Other research has also shown that AS events in the mammary gland of dairy cows are implicated in the host immune response [9]. Therefore, by identifying the specific DE mRNA isoforms produced via AS, the complexity of the transcriptome data may be reduced and may allow for the identification of specific mRNA isoforms that contribute to observed phenotypic variation. These specific mRNA isoforms could then be targeted in future SNP discovery studies, which could be implemented in future breeding programs involving marker - assisted or genomic selection.

Therefore, the goals of this study were to: (1) identify DE annotated mRNA isoforms between milk somatic cells (SC) samples from healthy and mastitic mammary quarters; (2) identify novel transcripts length from annotated genes or novel transcripts associated with non - annotated genes DE between healthy and mastitic samples; (3) identify functional candidate transcripts involved in immune processes that could potentially be associated with differences in the host response to mastitis that may be genomic track regions to target for future SNP discovery studies; and (4) annotate QTLs located in the coding regions of the identified DE mRNA isoforms.

## Results and discussion

### Global transcript expression

A total of 182 million single - end reads were generated from milk SC samples. RNA - Sequencing analysis revealed that 89.87% of these reads were mapped to the annotated bovine reference genome (ARS_UCD1.2; Table 1). The total number of transcripts expressed by milk SC from healthy and mastitic samples was 88,050 and 85,486, respectively (RPKM ≥ 0.2). To look at the functional relevance of these AS, we focused on the genes with two or more mRNA isoforms with at least one mRNA isoform being DE. In total, 333 DE transcripts were identified (FDR < 0.05 and |FC| > 2), each having at least 2 mRNA isoforms, with at least one being DE (Supplementary Table 1). These 333 DE mRNA isoforms corresponded to 303 unique genes. Among the 333 DE transcripts, 68 mRNA isoforms were annotated in the bovine reference genome (ARS.UCD1.2; Table 2), 249 were novel transcript lengths of known genes (Supplementary Table 2) and 16 were novel transcripts of non - annotated genes (Table 3), which were later annotated using NCBI blast (blastx; https://blast.ncbi.nlm.nih.gov/Blast.cgi). Of the 333 DE mRNA isoforms, 248 were under - expressed in the mastitic samples compared to the healthy samples (FC value < − 2); the most under - expressed mRNA isoform in the mastitic group compared to the healthy group was the bovine leukocyte antigen (*BOLA*) gene (FC = − 28,511.36). As discussed at length by Asselstine et al. [13], the *BOLA* genes are a complex group of genes and some of them are highly polymorphic. This in turn leads to variation in the animal’s ability to recognize antigens and carry out antigen presentation, making some animals more susceptible to disease and infection than others [14]. One of the reasons of BoLA being under-expressed could be the presence of polymorphisms or other functional variations that directly affects its expression, for example any polymorphisms impacting the amino acid and thus the formation of the protein. Alternatively, 85 isoforms were over - expressed in the mastitic samples compared to the healthy samples (|FC| > 2); the most over - expressed mRNA isoform was solute carrier family 36 member 1 (*SLC36A1*; FC = 3688.84). The solute carrier family in general is involved in the movement of amino acids [15]; and research has shown that amino acid transporters, such as the solute carrier family, affect T - cell fate decision, which has a central role in immune response [16].

**Table 1:** Alignment statistics of the 12 milk somatic cells samples collected from 6 Holstein dairy cows^a^

Group	Sample ID	Total reads mapped	Uniquely mapped reads%	Non - specifically mapped reads%	Unmapped reads%
Healthy	50A^b^	22,791,224	91.35	5.19	3.46
	50C^c^	12,757,084	81.84	7.50	10.67
	50E^d^	11,257,761	85.75	5.56	8.70
	50G^e^	9,041,681	78.70	4.87	16.43
	70A^f^	11,737,755	87.18	12.34	0.47
	70E^g^	16,124,047	95.17	4.36	0.48
		Total = 83,709,552	Av = 86.66	Av = 6.63	Av = 6.70
Mastitic	50B^b^	18,884,191	93.19	5.20	1.61
	50D^c^	26,510,003	92.70	6.47	0.83
	50F^d^	14,613,683	91.21	5.67	3.11
	50H^e^	15,174,409	90.40	6.86	2.75
	70C^f^	10,692,707	95.33	3.93	0.74
	70G^g^	12,365,761	95.63	3.95	0.41
		Total = 98,240,754	Av = 93.08	Av = 5.35	Av = 1.58

^a^Samples were aligned to the ARS_UCD1.2 bovine reference genome; ^b^These samples were collected from the same cow (3rd lactation, 74 DIM); ^c^These samples were collected from the same cow (2nd lactation, 44 DIM); ^d^These samples were collected from the same cow (3rd lactation, 178 DIM); ^e^These samples were collected from the same cow (2nd lactation, 133 DIM); ^f^These samples were collected from the same cow (2nd lactation, 7 DIM); ^g^These samples were collected from the same cow (1st lactation, 236 DIM)

**Table 2:** Differentially expressed mRNA isoforms from the 12 milk somatic cells from the 6 Holstein dairy cows with both gene and length previously annotated^a^

Feature ID	Position	Transcripts annotated	Transcript length	***P***-value	Fold change	FDR^**b**^
*ATF5*	18:56269037–56,273,031	5	1193	3.68E-06	− 1802.71	5.41E-03
*ATXN1*	23:40688295–40,829,586	6	2830	6.94E-07	− 4394.88	2.12E-03
*BTN1A1*	23:31585190–31,591,478	6	2691	3.66E-05	− 30.50	2.03E-02
*CCL20*	2:115948257–115,951,955	4	986	5.93E-05	81.84	2.86E-02
*CCN2*	9:69887188–69,890,613	5	2609	6.79E-06	− 1718.78	8.11E-03
*CCRL2*	22:52998332–53,000,232	3	1344	7.06E-05	15.52	3.17E-02
*CDC42BPB*	21:67623261–67,678,642	11	9648	1.07E-04	− 371.82	4.09E-02
*CSN1S2*	6:85529904–85,548,556	6	1198	8.12E-05	− 32.69	3.49E-02
*CSN3*	6:85645853–85,658,910	5	846	6.88E-05	− 26.13	3.12E-02
*CSN3*	6:85648339–85,658,926	5	768	1.92E-05	− 50.94	1.51E-02
*CYLD*	18:19137683–19,199,449	8	2911	1.45E-05	475.25	1.28E-02
*DHCR24*	3:91411765–91,445,037	12	3354	3.29E-05	− 650.78	1.91E-02
*DNMBP*	26:20826581–20,942,446	9	4857	6.55E-05	− 229.80	3.02E-02
*DSP*	23:47824434–47,868,337	3	9482	1.32E-04	− 31.07	4.49E-02
*EFHD1*	3:112396167–112,440,870	2	2513	6.47E-05	− 20.79	2.99E-02
*EFNB1*	X:81635594–81,648,094	3	2853	7.40E-05	− 534.58	3.27E-02
*ELF5*	15:65019975–65,052,826	9	2021	1.33E-04	− 49.35	4.49E-02
*ELF5*	15:65019975–65,065,633	9	1496	6.64E-07	− 234.40	2.10E-03
*ENSBTAG00000009049*	5:57433436–57,437,847	2	2326	7.23E-05	− 294.68	3.23E-02
*EPB41L2*	9:68956837–69,092,316	17	3068	1.30E-04	− 377.19	4.49E-02
*FCSK*	18:1644176–1,656,608	10	3249	1.38E-04	− 495.38	4.58E-02
*GLYCAM1*	5:25478786–25,482,857	4	1806	2.13E-05	− 40.39	1.59E-02
*GPRC5B*	25:17478831–17,498,791	2	1321	8.84E-05	− 49.93	3.68E-02
*GSE1*	18:11589111–11,644,469	9	3894	3.22E-05	− 1110.95	1.90E-02
*ITGB6*	2:36153834–36,247,251	4	3394	1.59E-04	− 631.00	4.94E-02
*KIAA1522*	2:120883699–120,890,243	10	3057	3.30E-06	− 1170.47	5.34E-03
*LENG8*	18:62878870–62,888,679	2	5021	2.20E-06	447.30	4.13E-03
*LHFPL2*	10:9538340–9,585,771	3	1298	2.63E-05	− 661.89	1.75E-02
*LIMK2*	17:70053292–70,092,008	12	1923	2.85E-06	401.17	4.83E-03
*LPO*	19:9202919–9,247,946	6	4658	1.27E-04	− 25.03	4.45E-02
*LTBP2*	10:85791791–85,897,975	16	5529	1.55E-05	− 931.09	1.34E-02
*LTF*	22:52952570–52,986,619	10	3112	3.11E-07	− 1768.47	1.51E-03
*MOB2*	29:50016711–50,043,414	7	1775	1.01E-04	− 258.89	3.97E-02
*MPP5*	10:79127656–79,234,049	11	2277	1.11E-04	− 211.97	4.16E-02
*MTSS2*	18:1488985–1,508,027	4	2177	1.26E-04	− 403.14	4.45E-02
*NFIX*	7:12486829–12,549,125	8	1387	1.15E-06	− 472.07	2.62E-03
*NR3C2*	17:9589541–10,018,758	3	2955	3.37E-05	− 92.14	1.93E-02
*NVL*	16:27418643–27,497,008	11	2559	4.01E-05	431.02	2.19E-02
*PAH*	5:66613983–66,732,963	10	1311	1.07E-04	− 134.03	4.09E-02
*PCBP4*	22:48973021–48,976,249	7	1212	1.58E-06	− 1886.75	3.06E-03
*PPARGC1A*	6:43380462–43,487,276	4	6323	8.79E-05	− 483.42	3.67E-02
*PRDM2*	16:53788898–53,921,908	11	7161	3.57E-05	− 174.89	2.01E-02
*PRKAA2*	3:89477898–89,549,744	2	1775	1.22E-04	− 105.23	4.35E-02
*PTPN14*	16:69036393–69,223,539	6	3747	6.36E-05	− 71.69	2.96E-02
*RALGAPB*	13:67377907–67,449,822	2	4672	2.22E-05	351.92	1.61E-02
*RASEF*	8:76173242–76,268,340	3	5985	7.85E-07	− 253.98	2.22E-03
*RBM27*	7:57417457–57,475,132	14	3259	2.38E-05	− 382.82	1.69E-02
*RERE*	16:44907739–45,028,328	13	3729	1.47E-07	− 7378.15	9.81E-04
*REXO1*	7:44150050–44,169,237	11	4370	5.96E-05	− 505.26	2.86E-02
*RGL2*	23:7466041–7,472,417	11	2288	5.92E-06	− 1232.16	7.36E-03
*RPS8*	3:101232559–101,235,265	5	1272	1.48E-05	− 899.12	1.29E-02
*SCAF11*	5:34252702–34,303,160	8	5366	4.29E-06	961.74	6.03E-03
*SFPQ*	3:110557887–110,571,478	6	2169	6.05E-06	− 683.60	7.43E-03
*SLC12A2*	7:25761570–25,853,058	6	4094	3.69E-06	− 295.71	5.41E-03
*SLC25A36*	1:128008637–128,042,419	13	2502	1.65E-09	1022.05	5.01E-05
*SLC34A2*	6:45185924–45,205,946	8	3949	4.76E-07	− 4234.58	1.87E-03
*SLC37A2*	29:28495548–28,524,597	9	4117	1.04E-05	707.73	1.10E-02
*TAF4*	13:55097811–55,161,568	9	3084	1.43E-04	− 167.37	4.67E-02
*TBC1D24*	25:1994690–2,006,139	11	4919	1.01E-04	− 28.37	3.97E-02
*TBKBP1*	19:38748409–38,762,591	8	1806	1.20E-05	− 849.58	1.19E-02
*TDG*	5:67640209–67,651,517	14	1233	1.50E-06	− 260.90	2.98E-03
*TNRC18*	25:38887201–38,970,458	13	11,628	6.69E-07	− 564.99	2.10E-03
*TNRC18*	25:38887201–38,970,458	13	11,364	5.46E-05	− 4688.21	2.71E-02
*TULP4*	9:94820215–94,960,850	8	4650	9.17E-07	− 3074.10	2.28E-03
*WAS*	X:86765549–86,772,520	11	1518	4.91E-05	− 161.16	2.48E-02
*ZBTB42*	21:69255817–69,256,846	3	1029	3.37E-05	− 682.55	1.93E-02
*ZDHHC24*	29:44577772–44,584,122	3	1128	3.69E-05	− 238.07	2.04E-02
*ZNF750*	19:49854333–49,863,765	2	3751	5.78E-05	− 33.30	2.84E-02

^a^Previosuly annotated in the ARS_UCD1.2 bovine reference genome; ^b^FDR: False discovery rate

**Table 3:** Novel mRNA isoform form the 12 milk somatic cells collected from 6 Holstein dairy cows and their predicted gene name

Category	Feature ID	Position	FDR^**a**^	Fold change	Predicted gene	Predicted species	Identity (%)	Pred.gene^**b**^ accession
Immune	*Gene_1149_1*	11:98497637–98,529,714	4.88E-05	−108.43	Maltose-binding periplasmic protein, Endoglin	*E. coli* K-12	61.18	5HZV_A
	*Gene_1149_8*	11:98510691–98,529,677	1.04E-06	−1312.31	Maltose-binding periplasmic protein, Endoglin	*E. coli* K-12	62.90	5HZV_A
	*Gene_593_7*	11:1185527–1,232,139	8.77E-05	36.05	ORF2 contains a reverse transcriptase domain	*Homo sapiens*	40.82	1VYB_A
	*Gene_2310_2*	3:86164085–86,182,808	3.12E-05	17.73	ORF2 contains a reverse transcriptase domain	*Homo sapiens*	36.17	1VYB_A
	*Gene_926_6*	11:62511669–62,519,754	1.07E-04	419.48	Protein Pellino Homolog 2	*Homo sapiens*	87.72	3EGB_A
	*Gene_2644_2*	11:6716314–6,728,323	1.17E-04	22.49	Crystal structure of an Interleukin-1 Receptor	*Homo sapiens*	89.13	3O4O_C
	*Gene_657_5*	11:6709199–6,716,867	1.17E-04	792.39	Crystal Structure of An Interleukin-1 Receptor	*Homo sapiens*	59.78	3O4O_C
	*Gene_1180_2*	11:99159315–99,178,645	1.41E-06	−3352.94	WD repeat-containing protein 34	*Homo sapiens*	89.47	6RLB_D
	*Gene_343_3*	6:115952167–115,960,221	4.28E-05	47.64	uS9	*Oryctolagus cuniculus*	43.55	6P4G_R
Normal cell function	*Gene_1007_5*	11:72505640–72,508,871	7.29E-05	228.43	Prolactin regulatory element-binding protein	*Homo sapiens*	73.58	5TF2_A
	*Gene_966_1*	11:71324276–71,361,339	7.86E-05	1401.95	Protein fosB	*Homo sapiens*	90.48	5VPA_A
	*Gene_726_6*	11:14696388–14,725,870	5.87E-05	−152.57	Spastin	*Homo sapiens*	82.09	6PEK_A
	*Gene_1335_4*	11:106231708–106,235,668	1.02E-04	−83.18	NADPH-cytochrome P450 reductase	*Saccharomyces cerevisiae*	55.81	3FJO_A
Unknown	*Gene_943_4*	11:66667335–66,667,877	6.67E-05	−230.47				
	*Gene_1711_9*	19:26666821–26,669,665	1.07E-04	−54.49				
	*Gene_1711_7*	19:26656544–26,669,665	1.28E-04	73.31				

^a^FDR: False discovery rate; ^b^Accession number of predicted gene using NCBI, which is the unique identifier for a sequence record. Novel mRNA isoform from the milk somatic cells of Holstein dairy cows had non - annotated genes in the ARS.UCD1.2 bovine reference genome and were later annotated with a predicted gene using NCBI blast (blastx; https://blast.ncbi.nlm.nih.gov/Blast.cgi)

### Functional analysis of known mRNA isoforms

#### Gene ontology (GO)

**Fig. 1:**
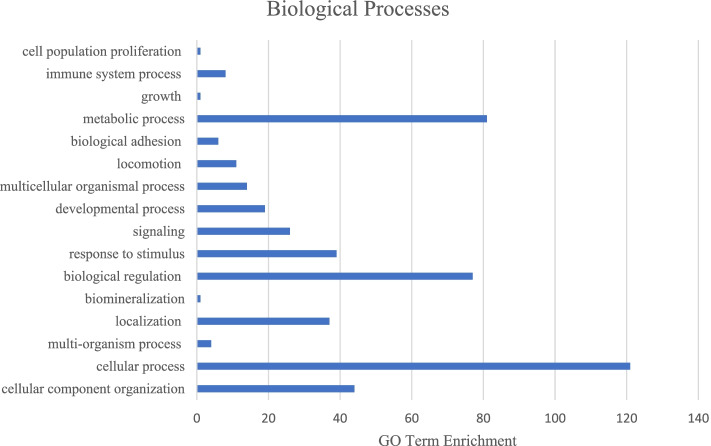
Associated Gene Ontology (GO) terms with differentially expressed genes in healthy and mastitic samples (FDR < 0.05, |FC| > 2) in the biological process GO category

**Fig. 2:**
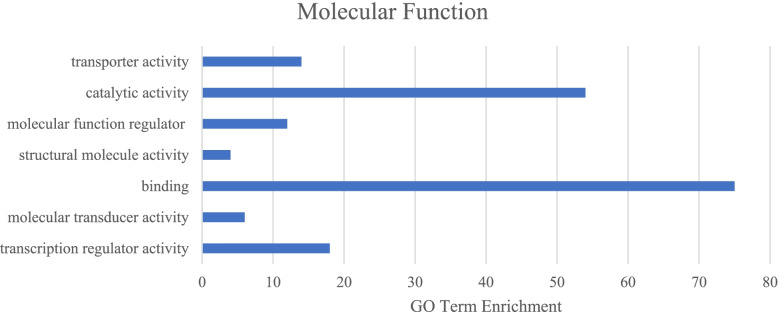
Associated Gene Ontology (GO) terms with differentially expressed genes in healthy and mastitic samples (FDR < 0.05, |FC| > 2) in the molecular function GO category

**Fig. 3:**
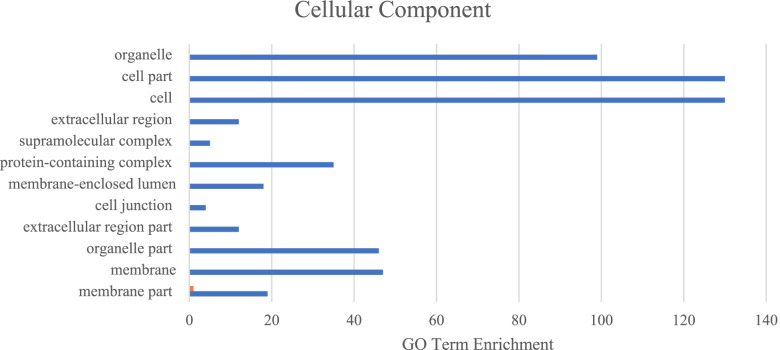
Associated Gene Ontology (GO) terms with differentially expressed genes in healthy and mastitic samples (FDR < 0.05, |FC| > 2) in the cellular component GO category

To ensure the most complete list of AS mRNA isoforms was used for functional analysis, the two groups with the gene name annotated in the ARS - UCD1.2 bovine reference genome were combined. This left 288 unique mRNA isoforms with gene name annotated. The three main GO categories (biological processes, molecular function, cellular process) were analyzed using the list of 288 mRNA isoforms. There were 16 significantly enriched GO terms were associated with the biological process GO category, with the most mRNA isoforms being involved with cellular process (*N* = 121) and metabolic process (*N* = 81; Fig. 1). For the molecular function GO category, the most enriched GO terms were binding (*N* = 75) and catalytic activity (*N* = 54; Fig. 2). Lastly, the most enriched GO terms for cellular component category were cell and cell part (both *N* = 130; Fig. 3). These results are in concordance with those found in Yang et al. [17] and Asselstine et al. [13], which both looked at the GO terms associated with mastitis in dairy cattle.

#### Gene network analysis

**Fig. 4:**
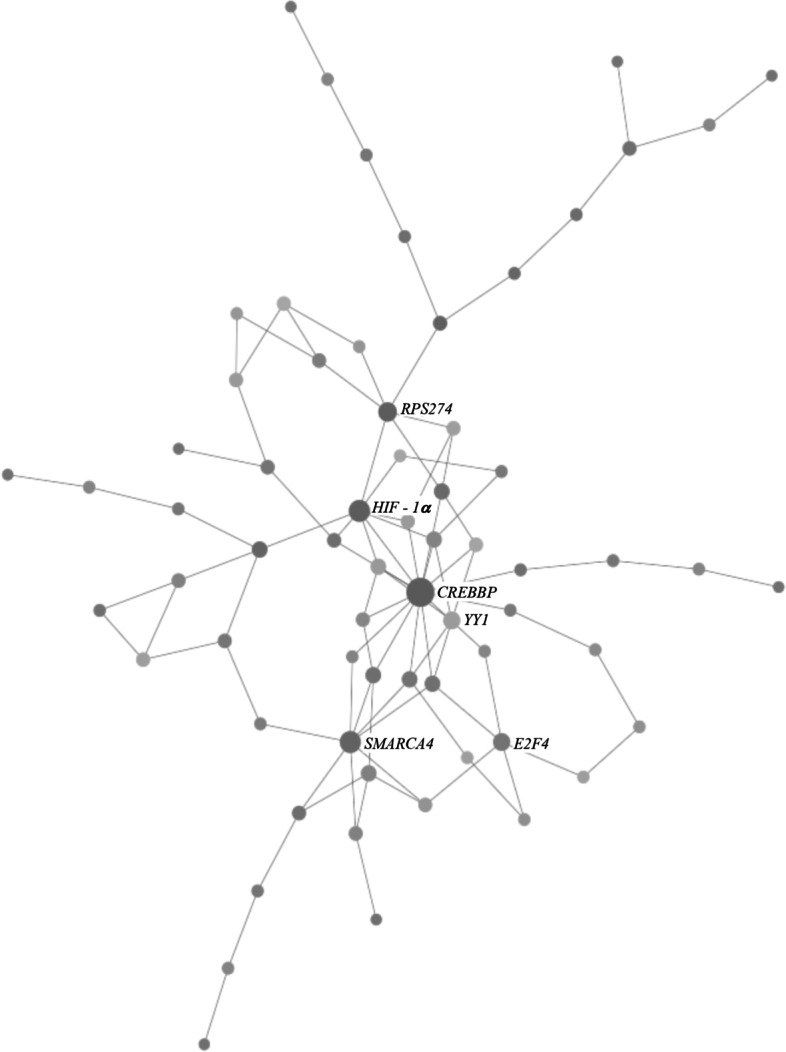
Gene network analysis constructed with the 288 unique mRNA isoforms Ensembl IDs that are involved in host immune response using NetworkAnalyst (http://www.networkanalyst.ca). The grey circles represent mRNA isoforms that are involved in the gene network analysis. The darker the grey and the larger the size of the circle represents its functional relevance, due to it being connected to numerous other mRNA isoforms

To complete the functional gene network analysis, the list of 288 combined and unique annotated mRNA isoforms was used. These 288 annotated mRNA isoforms were involved in 67 significantly enriched pathways including: regulation of cytokine biosynthetic process, positive regulation of cytokine secretion, cell - cell adhesion and immune response (Table 4). These gene networks are all connected via a few key nodes (cyclic AMP response element - binding protein binding protein (*CREBBP*), hypoxia inducible factor 1 subunit alpha (*HIF - 1α*), switch/sucrose non-fermentable related, matrix associated, actin dependent regulator of chromatin, subfamily A, member 4 (*SMARCA4*), ribosomal protein S27a (*RPS27A*); Fig. 4). Of these 4 key nodes, 3 of them are expressed in our list of DE mRNA isoforms (*CREBBP*, *HIF - 1α* and *SMARCA4*).

**Table 4:** Significant enriched metabolic pathways (FDR < 0.01) associated with the list of differentially expressed mRNA isoforms from the 12 milk somatic cell samples collected from the 6 Holstein dairy cows

Metabolic Pathway	FDR^**a**^	Total genes in pathway (n)	DE^b^ genes with mRNA AS variant in pathway (n)
Regulation of cytokine biosynthetic process	2.46E-08	708	17
Positive regulation of cytokine secretion	1.29E-06	1160	19
DNA replication initiation	5.27E-06	604	13
DNA_dependent DNA replication	1.99E-05	68	5
DNA damage response, signal transduction by p53 class mediator	2.16E-05	317	9
Aerobic respiration	2.63E-05	72	5
Neurotransmitter secretion	3.14E-05	13	3
Behavior	8.77E-05	48	4
DNA damage checkpoint	1.14E-04	487	10
Tyrosine phosphorylation of STAT protein	1.33E-04	232	7
Homeostasis of number of cells	1.47E-04	406	9
One_carbon metabolic process	1.64E-04	22	3
Positive regulation of hydrolase activity	1.97E-04	59	4
Cell maturation	2.11E-04	426	9
Positive regulation of transcription, DNA_dependent	3.41E-04	28	3
Dephosphorylation	3.54E-04	6	2
Regulation of protein phosphorylation	9.88E-04	90	4
Regulation of peptidyl_tyrosine phosphorylation	1.06E-03	41	3
Positive regulation of cysteine_type endopeptidase activity involved in apoptotic process	1.14E-03	42	3
Positive regulation of cytokine biosynthetic process	1.28E-03	11	2
Cell_cell adhesion	1.78E-03	49	3
Apoptotic nuclear changes	1.79E-03	178	5
DNA_dependent transcription, elongation	1.81E-03	106	4
Protein transport	2.41E-03	15	2
Gamete generation	2.74E-03	16	2
JAK_STAT cascade	2.97E-03	291	6
Regulation of cell migration	4.93E-03	1	1
Nitrogen compound metabolic process	5.08E-03	227	5
Ribosome biogenesis	5.18E-03	22	2
Regulation of protein metabolic process	5.87E-03	147	4
Cellular carbohydrate metabolic process	6.89E-03	79	3
Hormone secretion	7.38E-03	81	3
Immune response	8.32E-03	28	2
Positive regulation of transferase activity	8.96E-03	166	4
Actin filament organization	9.51E-03	30	2
Positive regulation of protein phosphorylation	9.84E-03	2	1
Hemostasis	1.01E-02	31	2
Calcium_independent cell_cell adhesion	1.47E-02	3	1
Negative regulation of translation	1.47E-02	3	1
Negative regulation of DNA binding	1.47E-02	3	1
Regulation of translational initiation	1.73E-02	41	2
Inorganic anion transport	1.90E-02	115	3
Endothelial cell proliferation	1.96E-02	4	1
Multicellular organismal development	1.98E-02	44	2
Steroid biosynthetic process	2.13E-02	215	4
Apoptotic mitochondrial changes	2.44E-02	5	1
Cell migration	2.44E-02	5	1
Regulation of phosphorylation	2.44E-02	5	1
MRNA metabolic process	2.80E-02	53	2
Proteolysis	2.92E-02	6	1
Actin polymerization or depolymerization	3.16E-02	140	3
Negative regulation of DNA replication	3.40E-02	7	1
Negative regulation of phosphorylation	3.40E-02	7	1
Synaptic transmission	3.42E-02	59	2
Lipid biosynthetic process	3.65E-02	374	5
Lysosomal transport	3.74E-02	62	2
Cell morphogenesis involved in differentiation	3.85E-02	63	2
Regulation of intracellular transport	3.96E-02	153	3
Monocarboxylic acid transport	4.31E-02	67	2
Negative regulation of transport	4.31E-02	67	2
Interleukin_2 production	4.36E-02	9	1
Regulation of gene expression, epigenetic	4.36E-02	9	1
Regulation of programmed cell death	4.36E-02	9	1
Actin filament_based process	4.49E-02	161	3
Nucleus organization	4.66E-02	70	2
Heme biosynthetic process	4.77E-02	165	3
Positive regulation of protein secretion	4.83E-02	10	1

^a^*FDR* False discovery rate; ^b^*DE* Differentially expressed

The first node *CREBBP* is 240 × under - expressed in the mastitic samples compared to the healthy samples. This binding protein activates specific transcription factors that are involved in cellular activities such as DNA repair, cell growth, differentiation and apoptosis by binding to the cAMP response element - binding protein (*CREB* [18]). The *CREB* is part of the innate immune system and has many roles in immune response, such as mediating the NF - κB– dependent antiapoptotic response in macrophages, thus macrophage survival and enhancement of the immune response [19, 20]. This is an important function of *CREB* as certain microbes or pathogens can induce apoptosis of macrophages as a mechanism to evade the host immune response [21]. One key aspect of determining the severity of the mastitis infection is how fast the host can adapt and clear the mastitis causing agents. Another function of *CREB* is that it induces *IL - 10* production, which is an inflammatory cytokine that has a key role in mediating the inflammatory loop to prevent unwanted tissue damage [22]. Tissue damage is another issue with mastitis as it reduces the number and activity of epithelial cells, contributing to a decreased milk production [23]. However, in our study, we did not find any DE mRNA isoforms associated with *IL - 10*. In summary, if *CREBBP* is significantly under - expressed or has a polymorphism preventing it from binding *CREB,* this could severely impact the host’s ability to kill the mastitis causing agents and reduce the inflammation and therefore, a case of mastitis that could have been minor becomes more severe.

The second node that explains the majority of the topology of the network is *HIF - 1α.* Hypoxia occurs when there is an oxygen shortage and has been shown to regulate innate immunological functions including apoptosis, phagocytosis of pathogens, antigen presentation, cytokine production among others [24]. As discussed in Palazon et al. [25], *HIF - 1α* is widely expressed and detected in virtually all innate and adaptive immune cell populations including macrophages [26], neutrophils [27], dendritic cells [28] and lymphocytes [29]. One study by Seagroves et al. [30] found that mice lacking *HIF - 1α* had impaired mammary differentiation and lipid secretion, which caused drastic changes in milk composition. Thus, illustrating that *HIF - 1α* plays a critical role in the function of mammary epithelium. To the best of our knowledge, this gene has not been directly linked with mastitis in ruminants but given its key role in the innate and adaptive immune function, as well as mammary epithelium, this may be a key gene in mammary gland health and function. In our study, this mRNA isoform was 1055 × under - expressed in our mastitic samples, therefore, further research should investigate if specific polymorphisms in this mRNA isoform that could impact its functionality.

Lastly, the *SMARCA4* gene proteins form one subunit of the switch/sucrose non - fermentable complex, which plays an important role in chromatin remodeling and is a known regulator for transcription and DNA repair [31, 32]. The *SMARCA4* gene is often mutated or silenced in tumors [33] and mutations in this gene have been associated with numerous human cancers such as small cell carcinoma of the ovary [34] and non - small cell lung cancer [32] among others. It has also demonstrated roles in T - cell development, T - cell lineage choice, T helper (Th) differentiation/function and macrophage function [34]. This is relevant because based on the type of mastitis pathogen in the udder, different T - cells are recruited [35]. However, to the best of our knowledge, no previous research has investigated the functions of *SMARCA4* regarding bovine mastitis, but it was 912 × under - expressed in the mastitic samples in this study, which suggests that this gene may be a candidate gene for further mastitis studies. Therefore, further research is needed to determine if this mRNA isoform could impact the host’s immune response or if it is just a key player in normal cell function.

### Functional analysis of novel mRNA isoforms

As previously discussed, we were not only interested in looking at mRNA isoforms previously annotated, but also novel mRNA isoforms generated by AS. We found that of the 333 DE transcripts, 16 were novel transcript lengths of unknown genes; out of these 6 were under - expressed in the mastitic samples compared to the healthy samples, while 10 were over - expressed. Using NCBI blast, the predicted gene name was identified for these novel transcripts of non - annotated genes in the ARS.UCD1.2 bovine reference genome. As presented in Tables 3, 13 of the 16 novel mRNA isoforms had a predicted gene associated with them. These 13 novel mRNA isoforms have been split into 3 separate categories: immune response, normal cell function and unknown function.

#### Immune response

The first two novel mRNA isoforms (*Gene_1149_1* and *Gene_1149_8*), are predicted to be Endoglin*.* Research has shown that the Endoglin gene product is associated with transforming growth factor - β (TGF - β) in humans and when there was a lack of expression of Endoglin in tumor cells, this correlated with poor clinical outcome [36]. Both novel mRNA isoforms were under - expressed in the mastitic group compared to the healthy group (FC = − 108.43, FDR = 4.88E-05; FC = − 1312.31, FDR = 1.04E-06, respectively), so they could potentially have a direct impact on the ability of the cow to react to the mastitic causing agents.

The next two novel mRNA isoforms (*Gene_593_7* and *Gene 2310_2*) were predicted to be associated with the open reading frame 2 (*OFR2*) of the Hepatitis E virus, which encodes the ORF2 viral capsid protein [37] and has biological processes associated with host - virus interaction. In general, mastitis is caused by bacterial and non - bacterial pathogens, but some research has shown that cows with clinical mastitis have other viral infections including infections bovine rhinotracheitis [38, 39] and foot - and - mouth disease [40]. Both isoforms were over - expressed in the mastitic samples (FC = 36.05, FDR = 8.77E-05; FC = 17.73, FDR = 3.12E-05, respectively) and therefore, it can be concluded that although this mRNA isoform does not directly deal with mastitis, it could make the animal more susceptible to other viral infections.

Novel mRNA isoform *Gene_926_6* is predicted to be the gene Pellino and this gene has been found to be expressed in various studies looking at immune response in humans [41] and mastitis in the goat mammary gland [42]. However, in neither of these studies was it the main focus so limited information could be found on its functionality in relation to mastitis or the immune system. In our study, this mRNA isoform was over - expressed in the mastitic samples (FC = 419.48; FDR = 4.09E-02).

The two novel mRNA isoforms (*Gene_2644_2* and *Gene_657_5*) were predicted to be associated with interleukin - 1 (*IL - 1)*, which is a pro - inflammatory cytokine and one of the elements of enhancing antigen recognition [43]. Interleukin - 1 helps to initiate the inflammatory response that can then be beneficial for initiating response to IMI, but it can also be damaging if its expression is excessive or prolonged. This is important for mastitis as the host needs to be able to recognize the antigen (or mastitis causing agent) quickly and efficiently, without causing more damage to the mammary tissue. In our study, both novel mRNA isoforms were over - expressed in the mastitic samples (FC = 22.49, FDR = 1.17E-04; FC = 792.39, FDR = 1.17E-04, respectively).

The WD repeat - containing protein 34 (*WDR34*) gene has been implicated in the immune response as a negative regulator of IL - 1R/TLR3/TLR4 - induced NF - κB activation pathway [44, 45]. This predicted gene is associated with the novel mRNA isoform *Gene_1180_2*, which was under - expressed (FC = − 3352.94, FDR = 2.95E-03) in the mastitic samples. As this AS variant is associated with a gene that is a regulator of the immune response, if it is not functioning properly, this could potentially impact the host’s response to the IMI.

Next, the predicted gene *uS9* associated with *Gene_343_3* was over - expressed in the mastitic samples compared to the healthy samples (FC = 47.64, FDR = 4.28E-05). One role of *uS9* is that it can block natural killer cell activation which play an important role in host defence. To the best of our knowledge, it has not been previously associated with mastitis so further research is needed.

#### Normal cell function

The novel mRNA isoform (*Gene_1007_5*) was predicted to be Prolactin, which is key in the maintenance of milk secretion [46]. It is not known if this specific mRNA isoform impacts mastitis but given the critical role of this gene in milk production it would be important to consider this isoform, which is 228x over - expressed in the mastitic samples (FC = 288.43, FDR = 3.24E-02).

Next, the predicted gene *FosB* was associated with the novel mRNA isoform (*Gene_966_1*) and was over - expressed in the mastitic samples (FC = 1401.95, FDR = 3.40E-02). The Fos family members are closely related with the Jun family members and both compose the AP - 1 transcription factor which participates in the control of cellular responses to regulate normal cell functions including cell death [47].

The novel mRNA isoform (*Gene_726_6*) associated with Spastin was under - expressed in the mastitic group (FC = − 152.57, FDR = 2.86E-02). Spastin is involved in microtubule dynamics for ATP - ase and therefore is important for normal cell function [48].

The predicted gene NADPH - cytochrome P450 reductase is critical for normal cell function and in this study was associated with *Gene_1335_4,* which was under - expressed in the mastitic group (FC = − 83.18, FDR = 3.99E-02). Although this gene was under - expressed, it is possible that the cause of this is due to the increased proportion of inflammatory cells in the mastitic milk SC [13].

#### Unknown function

Alternatively, three splice variants of the 16 novel transcripts of non - annotated genes did not have a predicted gene name and perhaps are extremely novel due to the inability for a match to be made in any species (*Gene_943_4, Gene_1711_9* and *Gene_1711_7*). Two AS variants were under - expressed (*Gene_943_4* and *Gene_1711_9*; FC = − 230.47, FDR = 6.67E-05 and FC = − 54.49, FDR = 1.07E-04, respectively) while one (*Gene_1711_7*) was over - expressed (FC = 73.31, FDR = 1.28E-04); due to not having a predicted gene name, no functional information could be identified for them. Thus, further research is required to determine the direct or indirect role they may have on mastitis or the immune system.

### QTL annotation

**Fig. 5:**
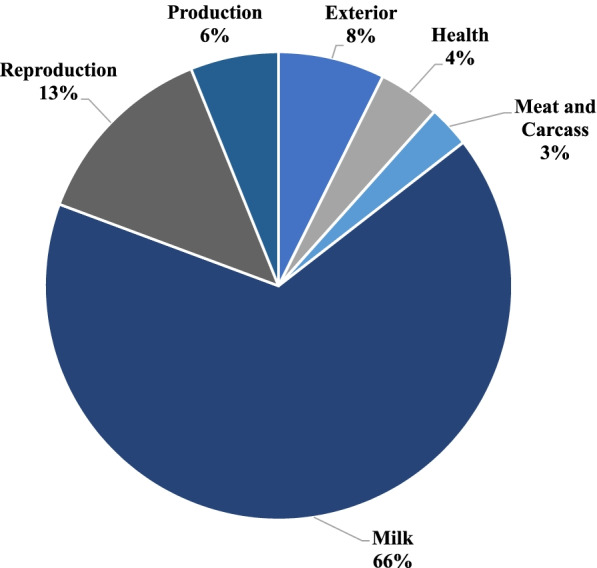
Categories of the previously annotated QTL overlapping within the list of DE mRNA genomic regions

Identifying QTL can make important connections between the phenotypic trait of interest and identify key differences in the host genome. The current cattle QTL database has 159,844 QTL relating to 653 different traits (release 42 [49]; https://www.animalgenome.org/cgi-bin/QTLdb/index). The QTL annotation was performed using the coordinates of the 333 AS DE mRNA isoforms (Supplementary Table 3). In total, 207 previously annotated QTL were located in the regions of DE mRNA AS variants (Supplementary Table 4). The QTL were annotated for milk (66%), reproduction (13%), exterior (8%), production (6%), health (4%) and meat and carcass (3%; Fig. 5).

As expected, due to the importance of milk production, the majority of QTL were associated with milk traits, with the largest amount of QTL being associated with milk protein percentage. Milk protein is a critical component of the bovine milk and in our study, 6 different mRNA isoforms (Supplementary Table 4) were associated with previously annotated QTL regions. One of these mRNA isoforms, Casein Kappa (*CSN3)*, has been identified in numerous studies. One study by Alim et al. [50] found *CSN3* to be an important candidate that influences milk production traits (i.e., milk protein) and could be used for the genetic improvement of milk production traits in dairy cattle. In our analysis, there were three different *CSN3* isoforms, *CSN3_1*, *CSN3_2* and *CSN3_5* that were 100x, 26x and 51x, under - expressed respectively, in the mastitic samples compared to the healthy samples, so close attention should be paid to these specific mRNA isoforms if implementing them into breeding practices.

When we look at the QTL associated with health traits, there are previously annotated QTL associated with somatic cell score (SCS), clinical mastitis (CM) and bovine respiratory disease (Supplementary Table 4). There are 9 previously annotated QTL corresponding to 5 different genes (solute carrier family 9 member A8 (*SLC9A8)*, lactoferrin (*LTF)*, ribosomal protein S6 kinase C1 (*RPS6KC1)*, Sad1 and UNC84 domain containing 2 (*SUN2)* and nuclear factor I X (*NFIX*)). These genes each have only one DE mRNA isoform associated with them. Both *SLC9A8_2* and *RPS6KC1_5* were over - expressed in the mastitic group (FC = 157.68 and 734.76, respectively). The *SLC9A8* gene is important in the protection of epithelial cells from bacterial adhesion [51, 52] and this is associated with the QTL trait of CM. The mRNA isoform *RPS6KC1_5* was annotated to be associated with SCS however, further research is needed to determine if it is a direct connection as literature is still scarce. Alternatively, 3 mRNA isoforms, *LTF_10*, *SUN_12* and *NFIX_8* were all under - expressed (FC = − 1768.47, − 85.03 and − 472.07, respectively) in the mastitic samples compared to the healthy samples. Lactoferrin_10 is associated with the QTL for CM and previous research has shown that *LTF* is a multifunctional protein with antimicrobial properties and plays an important role in innate immunity participating in the host first line defense [53, 54]. Interestingly, the mRNA isoform associated to *LFT,* was one of the most under - expressed in the mastitic samples compared to the healthy samples, which suggests this mRNA may be an important candidate gene to better understand the mechanisms involved in the development of mastitis. The *SUN2_12* and *NFIX_8* mRNAs isoforms are both associated with the QTL for SCS, however more research is needed to determine how these mRNA isoforms are related to mastitis before implementing them into breeding practices.

## Conclusion

The AS of known mRNA isoforms is significantly enriched in immune pathways such as cytokine secretion and cell - cell adhesion. Numerous novel mRNA isoforms were also identified that are involved with the immune system or mastitis. However, further research is needed to validate predicted genes and determine the exact impact they would have in relation to mastitis resistance. QTL annotation analysis revealed that the loci containing the identified DE mRNA isoforms overlap with QTL associated with CM and SCS, as well as milk traits including milk protein percentage and milk yield. In conclusion, LGRM identified novel mRNA isoforms that could lead to differences in the immune response of Holstein dairy cows. This research could aid in the implementation of breeding practices to aid in breeding healthier animals that are better able to adapt or prevent mastitis infections using either marker - assisted or genomic selection.

## Methods

### Animals and sample collection

This study was approved by the UC Davis Institutional Animal Care and Use Committee (IACUC). Sample collections and procedures were performed in accordance with the approved guidelines of UC Davis IACUC. The transcriptomes of 12 bovine milk somatic cell samples were characterized from 6 Holstein dairy cows using RNA - Sequencing to compare healthy and mastitic quarters within cows. Two different milk samples were collected from each cow, one sample from the mastitic quarter which was found using the California mastitis test and the other sample taken diagonally across from the mastitic quarter and classified as healthy (*N* = 12 [13]), based on having a SCC < 100,000 cells/mL. The cow’s teat was cleaned with gauze and damped in 70% isopropanol, then 50 mL of milk sample was taken from each quarter using a 3 cm plastic cannula (Genesis Industries Inc., Elmwood, WI) to ensure no bacteria contaminated the sample. Milk samples were kept on ice and immediately processed for RNA extraction using a Trizol reagent to preserve the integrity of the RNA.

### RNA extraction, library construction and sequencing

Transcriptomic analysis of 12 samples from bovine milk SC was performed using RNA - Sequencing technology as described by Cánovas et al. [55]. RNA sample preparation was also described in Cánovas et al. [55] and RNA quality was evaluated using the RNA integrity number (RIN) value from the Experion automated electrophoresis system (BioRad, Hercules, CA [56]). The RIN values ranged from 8.0 to 9.0 in all milk SC samples, indicating good RNA quality [56]. Library construction was performed using the TrueSeq RNA sample preparation kit (Illumina, San Diego, CA [57]). Sequencing was completed with an Illumina HiSeq 2000 analyzer that yielded 100 base pair (bp) single read sequences [13].

### Transcriptome analysis

#### Sequence assembly and quantification

Quality control, including the trimming of reads, was performed by CLC genomics workbench (CLC Bio Version 20.0.4, Aarhus, Denmark) using the quality trimming scores: limit = 0.05; maximum number of ambiguous bases = 2; discard reads below 100 bp. Trimming the reads allowed for single - end sequences to be included in this analysis, which improved the quality of the alignment sequences. After trimming, all samples passed the quality control analysis based on GC content, Phred score and over - represented sequence parameters as described by [55].

The trimmed sequences were aligned to the bovine reference genome (ARS_UCD1.2; ftp://ftp.ensembl.org/pub/release-100/) using CLC genomics workbench, with a Large Gap Read Mapping (LGRM) approach [58, 59]. The LGRM tool can map sequence reads that span introns without requiring prior transcript annotation, thus allowing the best match for a given read to be identified. The mapping criteria that followed included mismatch, insertion and deletion costs of 2, 3 and 3, respectively and was performed as described by Cardoso et al. [58].

Transcript discovery was performed to identify transcripts in both healthy and mastitic samples using CLC genomics workbench. We first performed transcript discovery on the healthy group which using the bovine reference genome and the LGRM assembly for the healthy group. Parameters for filtering include gene merging distance = 50, minimum reads in gene = 10 and minimum predicted gene length = > 200 bp [59]. For the mastitic transcript discovery, we used the predicted RNA and gene tracks generated from the healthy group and annotated bovine reference genome. Thus, the predicted mRNA file contained predicted information from both sets of samples (healthy and mastitic), in addition to annotated genome information, which was used as a reference track to map the reads of each sample. Transcript levels were quantified in reads per kilo base per million mapped reads (RPKM [57]). By normalizing the data for RNA length and total reads in each sample, the RPKM measure facilitated comparisons of transcript levels between groups [4]. A threshold of RPKM ≥0.2 was used to select transcripts expressed in each sample, as this threshold has previously been used by Wickramasinghe et al. [5] to detect gene expression in milk SC. We considered mRNA isoforms to be DE between healthy and mastitic samples when it had a false discovery rate (FDR) < 0.05 and a fold - change (|FC|) > 2.

#### Transcript annotation and functional enrichment analysis

Transcript annotation of the bovine mRNA isoforms was retrieved from the BioMart Database (http://useast.ensembl.org/biomart/martview/). Gene ontology (GO) enrichment analysis was performed using Panther software [60]. The GO terms associated with the three main GO categories such as biological processes, molecular function and cellular component were analyzed [61]. Gene network analysis was performed using NetworkAnalyst (http://www.networkanalyst.ca) software. NetworkAnalyst software performs meta - analysis on gene expression data sets to determine important features, patterns, functions and connections among genes [62–66]. A list of the Ensembl gene Ids related to DE mRNA isoforms was uploaded and the program’s default parameters were used.

For the novel mRNA AS variants, the predicted gene associated with each was identified. This was done using NCBI viewer to download the FASTA files based on the location (start and end position) of the novel transcripts in the bovine genome. These FASTA files were then uploaded to NCBI blast (blastx; https://blast.ncbi.nlm.nih.gov/Blast.cgi). The protein data bank protein feature was used as reference database to find similar sequences genome annotation in either bovine or in other species allowing inferences about a genes and functions to be made. From the predicted gene associated with each novel isoform further functional analysis was performed on a gene - by - gene manner.

### QTL annotation analysis

Lastly, QTL annotation analysis was performed using the R package: Genomic functional Annotation in Livestock for positional candidate LOci (GALLO) (https://CRAN.R-project.org/package=GALLO; [67]). The genome coordinates of the DE transcripts were used, as well as the QTL .gff annotation file retrieved from the cattle QTL Database (https://www.animalgenome.org/cgi-bin/QTLdb/index [49, 68]). We used windows of 1000 bp to account for 100 upstream and 100 downstream of each DE transcript [69].

## Supplementary Information

**Additional file 1.**

## Data Availability

The datasets generated and/or analyzed during the current study are available in the NCBI’s Gene Expression Omnibus repository, https://www.ncbi.nlm.nih.gov/geo/query/acc.cgi?acc. Accession ID = GSE131607.

## Abbreviations

AS: Alternative splicing
BP: Base pair
*BOLA*: Bovine leukocyte antigen
*CSN3*: Casein kappa
*CREB*: cAMP response element - binding protein
*CREBBP*: Cyclic AMP response element - binding protein binding protein
DE: Differentially expressed
FC: Fold change
FDR: False discovery rate
GO: Gene ontology
*HIF - 1α*: Hypoxia inducible factor 1 subunit alpha
*IL – 1*: Interleukin - 1
LGRM: Large gap read mapping
*LTF*: Lactoferrin
*NFIX*: Nuclear factor I X
*ORF2*: Open reading frame 2
*RIN*: *RNA integrity number*
*RPKM*: Reads per kilo base per million mapped reads
*RPS27A*: Ribosomal protein S27a
*RPS6KC1*: Ribosomal protein S6 kinase C1
RNA – Seq: RNA - Sequencing
SC: Somatic cells
*SLC36A1*: Solute carrier family 36 member 1
*SLC9A8*: Solute carrier family 9 member A8
*SMARCA4*: Switch/sucrose non-fermentable related, matrix associated, actin dependent regulator of chromatin, subfamily A, member 4
*SUN2*: Sad1 and UNC84 domain containing 2
*TGF - β*: Transforming growth factor - β
*WDR34*: WD repeat - containing protein 34
